# Exploring the intensity, barriers and correlates of physical activity In Iranian pregnant women: a cross-sectional study

**DOI:** 10.1136/bmjsem-2020-001020

**Published:** 2021-10-06

**Authors:** Katayon Ahmadi, Leila Amiri-Farahani, Shima Haghani, Seyedeh Batool Hasanpoor‑Azghady, Sally Pezaro

**Affiliations:** 1 Department of Reproductive Health and Midwifery, Iran University of Medical Sciences, Tehran, Iran; 2 Department of Biostatistics, Iran University of Medical Sciences, Tehran, Iran; 3 School of Nursing, Midwifery and Health, Centre for Arts Memory and Communities, Coventry University, Coventry, UK

**Keywords:** physical activity, pregnancy, quantitative

## Abstract

**Objectives:**

To determine the intensity, barriers and correlates of physical activity (PA) in Iranian pregnant women.

**Methods:**

This cross-sectional study was carried out with 300 eligible pregnant women referred to the Ilam health centres and bases using stratified random sampling with proportional allocation. Data collection tools included a demographic and obstetrical history questionnaire, the Pregnancy Physical Activity Questionnaire and the Exercise Benefits/Barriers Scale. The association between demographic and obstetrical characteristics and PA intensity and barriers were studied using multiple linear regression models.

**Results:**

The mean and SD of the total score of PA intensity were 47.15 and 26.25 metabolic equivalent of task (MET)-hour/week, respectively. The highest and the lowest barriers were related to the time expenditure (42.77±18.04) and family discouragement (50.72±24.99) constructs, respectively. The PA intensity was significantly associated with prepregnancy or early pregnancy body mass index (B=25.6), ethnicity (B=16.94), level of education (B=−8.77), number of children (B=5.95), gestational age (B=0.81), participation in childbirth preparation classes (B=−11.27), habitual exercise before pregnancy (B=6.09), income (B=−9.22). The PA barriers were significantly associated with ethnicity (B=−4.96), income (B=2.23) and habitual exercise before pregnancy (B =−1.35).

**Conclusion:**

PA intensity may be enhanced by encouraging individuals to be more physically active before pregnancy. Additionally, strategies to enhance support from friends and family to engage in PA throughout pregnancy and PA interventions focused on women with lower levels of income and education are required.

**Key messages**

**What is already known?**

- Despite all the known benefits of physical activity (PA), many pregnant women do not participate in regular PA.
- Previous studies have cited different barriers and facilitators to PA during pregnancy, including personal, environmental, organisational and political factors.
- Iranian women’s very low engagement in moderate to vigorous-intensity exercise during pregnancy, there are likely several context-specific barriers to PA.
- No study has addressed such barriers among Iranian pregnant women specifically for whom PA is considered highly important.

**What are the new findings?**

- In the present sample of Iranian pregnant women, the highest weekly energy expenditure was attributed to light-intensity PA.
- The highest and the lowest barrier scores were related to the time expenditure and family discouragement constructs, respectively.
- To increase PA’s intensity, it is best to encourage individuals to be more physically active before pregnancy and improve family and friends’ support to encourage women to exercise during pregnancy.
- Interventions should be developed that focus on pregnant women with low education and low income in improving the intensity of PA.

## Background

Pregnancy is a life-changing event that can alter individuals’ physical activity (PA).^1^ Yet, despite all the known benefits of PA, many pregnant women do not participate in regular PA.^2^ The US Department of Health and Human Services (2018) has recommended that all pregnant women who do not have specific contraindications participate in regular aerobic and strength-training exercises during pregnancy.^3^ PA during pregnancy is associated with reduced gestational diabetes, preterm labour and risk of pre-eclampsia^2^ and decreased postpartum depression.^4–6^ Moreover, exercise in the second and third trimesters lowers the risk of caesarean birth.^7^ Thus, improved strategies to engage pregnant women in PA would be welcomed.

One study conducted in the USA identified that 31% of pregnant women included as participants reported engaging in mild-intensity activities, 38% in moderate-intensity and 32% in vigorous-intensity PA.^4^ In contrast, a survey conducted with pregnant women in Isfahan (a city in Iran) indicated that 98.7% of pregnant women engaged in mild-intensity PA, while only 1.3% reached moderate-intensity levels.^5^ Previous studies have cited different barriers and facilitators to PA during pregnancy, including personal, environmental, organisational and political factors.^8–10^ However, given Iranian womens’ very low engagement in moderate to vigorous-intensity exercise during pregnancy, there are likely several context-specific barriers to PA. The low levels of PA noted are not necessarily caused by a lack of interest,^11^ and many of the barriers towards PA in general in this region may relate to religion and culture. For example, due to wearing a hijab outdoors, many Muslim women in the warmer climates of Iran may prefer to exercise indoors, which is not always available.^12^ Most Iranian women identify as housewives, have no income and thus depend on their partners to pay for PA.^13^ This lack of financial independence may also be an important barrier to undertaking PA. Conversely, while limited entertainment is available for women in Iran, a high desire to watch television series is considered another obstacle towards PA.^14^ Finally, a lack of awareness and/or knowledge on how exercise can be incorporated into everyday life without additional cost has also been identified as a barrier to engaging in exercise among individuals in this context.^15 16^

While the number of studies outlined here relate to barriers towards PA for Iranian women, no study has addressed such barriers among Iranian pregnant women specifically for whom PA is considered highly important. Thus, a cross-sectional study to identify the most important PA-associated factors for pregnant Iranian women was considered essential for designing future interventional studies to increase PA and improve maternal health. Accordingly, this study determined the intensity, barriers and correlates of PA among Iranian pregnant women.

## Methods

### Study design

The present cross-sectional study included pregnant women referred to perinatal clinics and health centres in Ilam, a province of Iran (see online supplemental material 1). The Health centre base is a subset of the comprehensive urban health centres located in the city’s suburbs. Informed written consent was obtained from all participants after providing information on the study purpose and procedures. Participants were assured of the confidentiality of their information.

10.1136/bmjsem-2020-001020.supp1 Supplementary data

### Study sample

We used G * power software to determine the required sample size. The sample size calculation yielded a required number of 300 participants, based on a 95% confidence level, a power of 0.8, an effect size of 0.06 and the consideration of 12 predictor variables.

Sampling was conducted continuously from September to December 2018 using stratified sampling from women referred to perinatal clinics by the proportional allocation of comprehensive urban health centre floors and bases. The inclusion criteria were Iranian, aged between 18 and 45 years, gestational age between 10 and 37 weeks, no contraindication to exercise during pregnancy,^17^ no movement restrictions, the ability to read and write, and informed written consent to participate in the study.

### Outcome measures and measurements

Data collection tools comprised a demographic and obstetrical history questionnaire (independent variables), the Pregnancy Physical Activity Questionnaire (PPAQ) and the Exercise Benefits/Barriers Scale (EBBS) (dependent variables).

The demographic and obstetrical history questionnaire consisted of variables such as age, prepregnancy or early pregnancy body mass index (BMI), ethnicity, level of education, occupation, income (reported based on individual’s perception of income and to what extent it meets individual’s living needs), number of previous pregnancies, number of children, gestational age, participation in childbirth preparation classes and the presence of habitual exercise before pregnancy.

The PPAQ designed by Chasan-Taber *et al*. assesses PA levels during pregnancy. This questionnaire asks respondents to select the category that best approximates the amount of time spent in 32 activities, including household/caregiving, occupational, sports/exercise and inactivity during the current trimester. At the end of the PPAQ, an open-ended section allows the respondent to add additional activities not listed. The duration of time spent in each activity is multiplied by its intensity to measure average weekly energy expenditure (MET-hour/week) attributable to each activity. Finally, the activities are divided into seven categories: sedentary activity, light-intensity, moderate-intensity, vigorous-intensity, household/caregiving, occupational and sports/exercise. The questionnaire′s reliability was confirmed with a Cronbach’s alpha of 0.78 for the total score and 0.87–0.93 for questionnaire categories.^18^ The validity and reliability of the Persian version of PPAQ were confirmed by Fathnezhad Kazemi *et al*^19^

The EBBS assesses the benefits and barriers of exercise. The English version of the EBBS consists of 43 items: 29 items of the benefits subscale and 14 of the barriers subscale. The scales are designed based on a 4-point Likert scale: strongly agree,^4^ agree,^3^ disagree,^2^ strongly disagree.^1^ The barrier scale is composed of four constructs: time expenditure (three items with total score 3–12), exercise milieu (six items with total score 6–24), physical exertion (three items with total score 3–12) and family discouragement (two items with total score 2–8). The minimum score for the barrier subscale is 14, while the maximum score is 56. In this scoring system, a higher score represents a greater perception of barriers. The internal reliability of the EBBS scale was confirmed with the Cronbach’s alpha coefficient of 0.952, and the benefits and barriers subscales were 0.953 and 0.886, respectively.^20^ The validity of the Persian version of the questionnaire was confirmed, and the reliability of the barriers subscale was validated with Cronbach’s alpha coefficient of 0.82 and Spearman Brown’s coefficient of 0.74.^21^ In this study, to examine the face validity of the PA barrier items, the questionnaire was completed by 20 eligible women of the study, according to whose opinions items 6 and 19 were repetitive. Therefore, due to the high correlation between these two items (r=0.87), item 6 (exercise makes me tired) was removed.

### Analyses

The data were analysed using SPSS V.21 (SPSS). Following the assessment of skewness and kurtosis, the quantitative data were considered to be normally distributed. Descriptive statistics, including frequencies and percentages, mean and SD, were used for describing demographic and obstetrical history variables, barriers to PA and intensity of PA. To compare the constructs of PA barriers, the obtained scores were normalised to a maximum score of 100. Higher scores indicate a higher number of barriers. To calculate each construct’s normalised score, its score was subtracted from the minimum score of that construct and divided by the difference of maximum and a minimum score of that construct. Finally, the answer of the obtained was multiplied by 100.

To compare the intensity and barriers of PA (quantitative variables) among demographic and obstetrical history variables (categorical variables), an independent t-test and ANOVA were used. Pearson’s correlation coefficient test was used to determine the relationship between the intensity and barriers of PA with demographic and obstetrical history variables that were considered quantitative variables.

To determine the relationship of each one of the independent variables (demographic and obstetrical history variables) on the dependent variable (intensity and barriers to PA separately as both were reported as quantitative variables), those variables that confirmed significance in the bivariate test (p<0.05) were entered into a multiple linear regression model using a backward strategy. Before the multivariate analysis, regression assumptions, including normality of residuals, homogeneity of residual changes, alignment of outliers and residuals independence, were examined and confirmed. Results from the linear regression analysis are presented as beta coefficients with associated 95% CIs. The level of statistical significance was set at p<0.05.

## Results

### Participants

**Figure 1:**
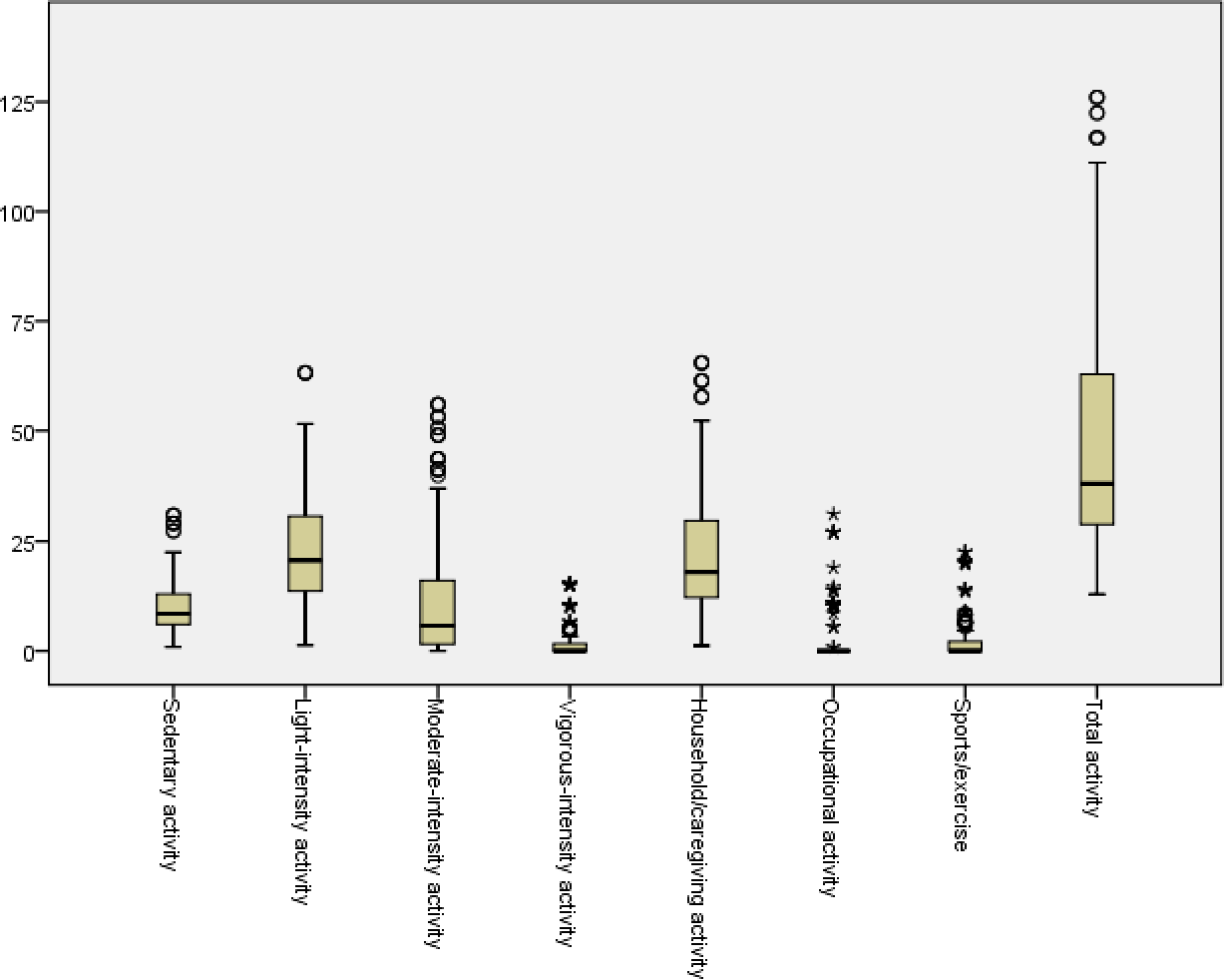
Box plot of scores of pregnant women’s PA intensity based on energy expenditure (MET-hour/week) and its categories (n=300). MET, metabolic equivalent of task; PA, physical activity.

Participants had a mean age of 27.52 (SD ±5.28) years. The mean prepregnancy or early pregnancy BMI was 26.03 (SD ±4.28) kg/ m^2^, and the gestational age was 23.77 (SD ±8.61) weeks. Most participants (n=181; 60.33%) had a university education and a favourable economic status (n=150; 50%). Box plot of scores of pregnant women’s PA intensity based on energy expenditure (MET-hour/week) and its categories are reported in figure 1.

### PA levels

The average total score of PA intensity in women was 47.15 (SD=26.25) MET-hour/week. The lowest amount of PA based on energy expenditure was attributed to vigorous-intensity PA. The highest amount of PA based on energy expenditure was attributed to light-intensity PA. The activities reported were mostly light-intensity and related to household/caregiving activities, while sports/exercise, vigorous-intensity activity and occupational activity were reportedly low (figure 1). Based on the activity type, household activities had the highest, while occupational activities had the lowest energy expenditure.

### PA barriers

The average total score in relation to PA barriers in this population was 30.72 (SD=5.81). Table 1 shows constructs of PA barriers, where the highest barrier scores were related to family discouragement and the lowest barrier scores with time expenditure constructs based on average % of the total score.

**Table 1:** Scores of pregnant women’s PA barriers and its constructs (n=300)

Constructs of PA barriers	Average score ±SD	Average % of total score ±SD
The total score	30.72±5.81	45.43±14.9
Time expenditure	6.85±1.62	42.77±18.04
Physical exertion physical exertion	4.73±1.32	45.50±22.08
Family discouragement	5.04±1.49	50.72±24.99
Exercise milieu	14.09±2.91	44.98±16.21

PA, physical activity.

Online supplemental materials 2 and 3, respectively, present the numerical indices total PA intensity, PA intensity and PA barriers regarding demographic and obstetrical history variables of the studied samples.

### PA correlates

To estimate the effect of demographic and obstetrical history variables on PA’s intensity and barriers, all variables with p<0.05 based on the results of online supplemental materials 2 and 3 were entered into the linear regression model using the backward method. According to the results in table 2, the relationship of demographic and obstetrical history variables on the intensity of PA, among the variables that entered into the model including prepregnancy or early pregnancy BMI, ethnicity, education, income, number of children, gestational age, participation in childbirth preparation classes and habitual exercising before pregnancy remained in the model. For one score increase in the prepregnancy or early pregnancy BMI in the obese category and increasing gestational age, the intensity of the PA increased by 25.6 and 0.81 units, respectively. The intensity score of PA in women who habitually exercised before pregnancy increased by 6.09 units. In women from the Fars ethnic grouping compared with those from the Kurdish grouping, there was a 16.94 score increase in the intensity score of PA and a 5.95 score increase for the addition of each child. At the same time, women with a high school diploma were reported to have an 8.77 lower score than those with a university education. Women who participated in childbirth preparation classes had an 11.27 lower score than those who did not. Women with 20–40 million rials income compared with those earning 40–100 million rials had a 9.22 lower score in the intensity of their PA. Consequently, 26.1% of the variations in the dependent variable (intensity of PA) was justified by the independent variables.

**Table 2:** Relationship of pregnant women’s demographic and obstetrical history characteristics with the PA intensity based on the results of multiple linear regression analysis

Independent variables		Unstandardised coefficients B	Standardised coefficient beta	95% CI for B	P value*	R^2^
Prepregnancyor early pregnancy BMI (kg/m^2^)	Underweight	Reference category				0.261
	Normal	8.63	0.16	−0.63 to 23.9	0.269	
	Overweight	10.82	0.2	−4.38 to 26.1	0.162	
	Obese	25.6	0.36	9.35 to 41.81	**0.002**	
Ethnicity	Fars	16.94	0.12	1.81 to 32.06	**0.028**	
	Lur	−8.24	−0.08	−18.66 to 2.18	0.121	
	Lak	2.96	0.02	−13.23 to 19.17	0.719	
	Kurdish	Reference category				
Level of education	Secondary	−5.24	−0.05	−16.80 to 6.31	0.372	
	Diploma	−8.77	−0.15	−15.10 to −2.44	**0.007**	
	University education	Reference category				
No of children		5.95	0.15	1.52 to 10.38	**0.009**	
Gestational age (weeks)		0.81	0.26	0.40 to 1.22	**<0.001**	
Participation in childbirth preparation classes	Yes	−11.27	−0.13	−21.28 to −1.25	**0.028**	
	No	Reference category				
A habit of exercising before pregnancy	Yes	6.09	0.11	0.46 to 11.73	**0.034**	
	No	Reference category				
Income (millions of rials)	Undesirable <20	3.86	0.04	−6.70 to 14.42	0.473	
	Fairly favourable: 20–40	−9.22	−0.17	−15.28 to −3.16	**0.003**	
	Optimal: 40–100	Reference category				

*Significance level: p<0.05, bold entries are significant results and are related to statistical tests.

BMI, body mass index; PA, physical activity.

According to the obtained results displayed in table 3, the relationship of demographic and obstetrical history variables on the barriers of PA, among the variables entered in the model including ethnicity, income, number of pregnancies, and habitual exercise; the number of pregnancies was the only variable that did not remain in the model. Women within the Lur ethnic grouping compared with those within the Kurdish ethnic grouping scored 2.52 more units, while women with an income of 20–40 million rials compared with women earning 20–40 million rials scored 2.23 more units in relation to the barriers to PA. Women who habitually exercised before pregnancy scored 1.35 fewer units in relation to the barriers to PA than women who did not. Overall, 8.3% of variations in the barriers to PA have been explained by independent variables, ethnicity, income and habitually exercising before pregnancy.

**Table 3:** Relationship of pregnant women’s demographic and obstetrical history characteristics with the PA barriers based on the results of multiple linear regression analysis

Independent variables		Unstandardised coefficients B	Standardised coefficient beta	95% CI for B	P value*	R^2^
Ethnicity	Fars	−0.34	−0.01	−3.79 to 3.11	0.845	0.083
	Lur	2.52	0.11	0.14 to 4.91	**0.038**	
	Lak	−4.96	−0.14	−8.78 to −1.14	**0.011**	
	Kurdish	Reference category				
Income (millions of rials)	Undesirable <20	0.40	0.02	−1.76 to 2.58	0.713	
	Fairly favourable: 20–40	2.23	0.19	1.84 to 3.62	**0.002**	
	Optimal: 40–100	Reference category				
No of pregnancies		0.39	0.05	−0.38 to 1.16	0.324	
A habit of exercising before pregnancy	Yes	−1.35	−0.11	−2.70 to −0.007	0.049	
	No	Reference category				

*Significance level: p<0.05, bold entries are significant results and are related to statistical tests.

PA, physical activity.

## Discussion

Our findings outline the intensity, barriers and correlates of PA in Iranian pregnant women. Here, the mean total score of PA intensity based on MET-hour/week and the SD were 47.15 and 26.25, respectively. The highest intensity PA was attributed to light-intensity activity. In contrast, the lowest intensity PA was attributed to vigorous-intensity activity. In relation to activity type, household activities had the highest, while occupational activities had the lowest energy expenditure overall.

The findings presented here are comparable to those of Nascimento *et al*^22^ except for the lowest activity level, which is related to exercising rather than occupational activity. Yet interestingly, in Nascimento *et al*^22^’s study, sampling was conducted during the postpartum period when it seems unlikely that women would engage in any exercise activity. Additionally, the number of employed women in the sample was estimated at 54.15%, indicating a sample experiencing increased occupational activities. While conversely, in this study, only 13.66% of participants were employed as physicians (7.3%), other employees (53.65%) and teachers (39.02%), respectively. Given that these jobs are generally associated with less energy expenditure, they may lower the level of occupational activity and energy expenditure. According to further studies, with increasing gestational age and increased participation in leisure time, PA also persists in decreasing well into the postpartum period.^23 24^ Such findings may point to the need for PA promotion in the workplace and throughout the postnatal period.

In another study, the lowest activity level was related to sport/exercise,^25^ while the lowest activity level was related to occupational activity in this study. Yet in Antosiak-Cyrak and Demuth’s study, one out of three women were urban residents, and the rest lived in rural regions,^25^ while in this study, all participants lived in the city. This suggests that PA promotion may need tailoring to address the needs of women living in both rural and urban settings, particularly as women living in urban areas can have greater access to sports facilities (such as clubs) than women living in rural areas, and therefore, engage in less exercise overall.^26^ Yet, these women may be more physically active in other ways. So it will also be important for future studies to capture qualitative data to offer richer context in this regard.

According to the results presented in this study, family discouragement was perceived as the greatest barrier to PA, while time expenditure was perceived as the least important barrier. Factors such as not having a sexual partner and family support have previously been associated with poor PA in women.^27^ Also, having an active spouse before pregnancy has been identified as the strongest predictor of performing moderate-intensity to vigorous-intensity PA during pregnancy.^28^ In another study, pregnant women also describe the value of social support from family, friends, and health professionals as facilitators to PA.^29^ Such findings indicate the need to engage families and spouses in future interventions designed to enhance PA in pregnancy.

Demographic and obstetrical history characteristics, prepregnancy or early pregnancy BMI, ethnicity, level of education, income, number of children, gestational age, participation in childbirth preparation classes and having exercise habits before pregnancy had a significant relationship with the total PA intensity. Accordingly, these characteristics will also be important to consider in the development of any future interventions. Specifically, women with a BMI above 30 kg/m^2^ before or at the beginning of their pregnancy had a higher mean energy expenditure intensity. Despite this finding, Haakstad *et al*^30^ have previously reported no significant relationship between the prepregnancy or early pregnancy BMI of women who exercise in the third trimester and women who did not. Other studies have similarly shown no significant relationship between prepregnancy BMI and exercise during pregnancy.^22 31^ This discrepancy may be explained by the fact that many of the participants in this study with a BMI above 30 kg/m^2^ had experienced more pregnancies than those with a lower BMI. Nevertheless, larger sample sizes in future may explain this phenomenon in greater clarity.

Although the intensity of energy expenditure in this study was higher in women who had a high BMI before or early pregnancy, this energy was predominantly spent on low-intensity activities. Consistent with these results, an earlier study also identified a statistically significant relationship between prepregnancy BMI in women who exercised in the third trimester and women who did not.^30^ Yet alternative studies have reported no significant relationships between prepregnancy BMI and exercise during pregnancy.^22 31^ Nevertheless, there remains an opportunity to promote higher intensity PA in this group, both before and after pregnancy.

In this study, the highest to lowest total energy expenditure belonged to the following ethnic groupings, respectively; Fars, Lak, Kurdish and Lur. This may be explained by the fact that Persian women may be more accustomed to exercise before pregnancy. For example, in our sample, 7 out of 11 Persian women (63.6%) were accustomed to exercising before pregnancy, while out of a population of 289 non-Persian women, only 103 (35.6%) of them exercised before pregnancy. Indeed, other research has established that habitually exercising before pregnancy increases the chance of exercising during pregnancy,^22^ and a lack of PA before pregnancy has been identified to be the strongest predictor of reduced exercise during the third trimester of pregnancy.^22 23 30^ More research is required to understand any links between ethnicity and PA more comprehensively. Nevertheless, there remains an opportunity for future interventions to promote PA before pregnancy in all populations, given the number of women from various ethnic groups not engaged in habitual PA during this time.

Notably, women with a university education experienced a higher intensity of PA. Lack of university education has previously been associated with physical inactivity in pregnant women elsewhere.^22 23 32^ Yet conversely, in Portuguese women, no statistically significant relationship between education and PA in pregnancy was found.^5 26^ Such findings suggest that targeted PA toward those with lower levels of education may be useful and that further evidence is required in this area. According to the current study, a higher income was also associated with increased energy expenditure. In two other studies, lower-income levels have similarly been associated with decreased PA.^4 33^ This suggests that those with lower incomes may also be usefully targeted in the future promotion of PA during pregnancy.

Those with a higher number of children endured a higher level of energy expenditure through carrying out light and moderate household/caregiving. At the same time, childless pregnant women had a higher energy expenditure for carrying out occupational, sport and vigorous activities. The findings of Chasan-Taber *et al*^31^ corroborate those presented here in this regard. Such findings may usefully inform the development of interventions promoting PA during pregnancy as women both begin and grow their families.

In our study, gestational age also had a significant correlation with energy expenditure. Indeed, previous studies suggest that participation in PA can increase significantly in the first trimester of pregnancy compared with the second and third trimesters of pregnancy.^33^ These findings may be useful in tailoring the promotion of PA throughout the entire duration of pregnancy. Consistent with this study’s results, no statistically significant correlation was found between age and PA.^4 26^ While in other studies, there has been a significant, positive correlation between age and exercise identified.^23 34^ These discrepancies indicate the need for larger samples and more nuanced mixed-methods explorations of PA, age and pregnancy overall.

This study found that women who did not attend childbirth preparation classes consumed more energy. In interpreting this relationship and perhaps contrary to expectations, childbirth preparation classes offered to pregnant women in Iran do not provide training on the importance of PA in pregnancy and its safety. As such, opportunities remain in integrating PA promotion in childbirth preparation classes in this context, particularly in relation to higher intensity PA. Yet, it is of note that the 85.29% of participants who did not attend childbirth preparation classes had a university education, as this may somewhat account for the higher levels of PA noted in this sample.

The total score of barriers to PA was significantly correlated with individual and obstetrical histories and certain characteristics, including ethnicity, income, number of pregnancies and the presence of habitual exercise before pregnancy. Having a lower income was also significantly correlated with the total score of barriers to PA. Other studies also reported low income as an important barrier,^35^ while others found no significant relationship.^26^ Such discrepancies may be explained by the inclusion of different study populations and suggest a need to achieve clarity through mixed-methods triangulation in this regard.

In our study, the number of pregnancies was significantly correlated with PA barriers. For example, women experiencing their third and fourth pregnancies reported greater barriers than those experiencing their first and second pregnancies. Contrary to the results of other studies,^23 36^ women pregnant for the first time with lower levels of education were more likely at risk of experiencing barriers to PA. This suggests that barriers to PA in all pregnancies could be usefully identified and addressed.

Not being used to exercise before pregnancy also had a significant correlation with barriers to PA. In line with the current study, pregnant women who had been inactive before pregnancy gained a higher barrier score elsewhere.^37 38^ As compulsory occupational activities can increase daily PA,^26^ habitual PA could usefully be promoted for women devoid of these before, during and after pregnancy.

A key strength of this study is that it has included multicentre populations and has evaluated the intensity and barriers of PA in all three trimesters of pregnancy. As it was undertaken with women experiencing low-risk pregnancies in urban areas, results cannot be generalised to wider populations. Future studies may usefully be conducted with women experiencing low-risk pregnancies or/and women experiencing high-risk pregnancies living in rural areas who can safely engage in PA. The Barriers to Physical Activity in Pregnancy Scale^39^ may usefully identify barriers to PA in pregnancy for all groups in future.

## Conclusion

This study has determined the intensity, barriers and correlates of PA in Iranian pregnant women. Encouraging individuals to be more physically active before pregnancy and enhanced support from family and spouses who incites women to exercise during and after pregnancy may increase the intensity of PA most effectively. PA interventions could usefully target pregnant women with lower levels of education and income specifically.

## Data Availability

Data may be obtained from a third party and are not publicly available. no comment.

## References

[1] Borodulin KM, Borodulin KM, Borodulin KM, et al. Physical activity patterns during pregnancy. Med Sci Sports Exerc 2008;40:1901–8. 10.1249/MSS.0b013e31817f1957 18845974 PMC3319731

[2] Gaston A, Vamos CA. Leisure-Time physical activity patterns and correlates among pregnant women in Ontario, Canada. Matern Child Health J 2013;17:477–84. 10.1007/s10995-012-1021-z 22488158

[3] USDHHS UDoHHS. Physical activity guidelines for Americans. Advisory Committee report 2018 2018.

[4] Marshall ES, Bland H, Melton B. Perceived barriers to physical activity among pregnant women living in a rural community. Public Health Nurs 2013;30:361–9. 10.1111/phn.12006 23808861

[5] Bahadoran P, Mohamadirizi S. Relationship between physical activity and quality of life in pregnant women. Iran J Nurs Midwifery Res 2015;20:282–6. 25878709 PMC4387656

[6] Summerbell CD, Summerbell CD, Summerbell CD, et al. The association between diet and physical activity and subsequent excess weight gain and obesity assessed at 5 years of age or older: a systematic review of the epidemiological evidence. Int J Obes 2009;33 Suppl 3:S1–92. 10.1038/ijo.2009.80 19597430

[7] Domenjoz I, Kayser B, Boulvain M. Effect of physical activity during pregnancy on mode of delivery. Am J Obstet Gynecol 2014;211:401.e1–401.e11. 10.1016/j.ajog.2014.03.030 24631706

[8] Cramp AG, Bray SR. A prospective examination of exercise and barrier self-efficacy to engage in leisure-time physical activity during pregnancy. Annals of Behavioral Medicine 2009;37:325–34. 10.1007/s12160-009-9102-y 19499279

[9] Irehovbude J, Ikhioya G, Okonigene D, et al. Perception of the benefits and barriers to antenatal exercise among pregnant women in Benin City, Nigeria. Proceedings of the International Conference on Bioinformatics & Computational Biology (BIOCOMP); 2018: The Steering Committee of The World Congress in Computer Science, Computer 2018:99–104.

[10] Evenson KR, Evenson KR, Evenson KR, et al. Perceived barriers to physical activity among pregnant women. Matern Child Health J 2009;13:364–75. 10.1007/s10995-008-0359-8 18478322 PMC2657195

[11] Andreasen AR. Marketing social change. California, USA: Jossey-Bass, 1995.

[12] Eldoumi H, Gates G. Physical activity of Arab Muslim mothers of young children living in the United States: barriers and influences. Ethn Dis 2019;29:469–76. 10.18865/ed.29.3.469 31367167 PMC6645718

[13] Statistical Center of Iran. Rates of working men and women in 2015. Available: https://www.amar.org.ir/Portals/0/PropertyAgent/5276/Files/6898/IG_niru3_94-1.pdf

[14] Ahmadi Tabatabaei SV, Ahmadi Tabatabaei SV, Ahmadi Tabatabaei SV, et al. Promoting physical activity in Iranian women: a qualitative study using social marketing. Electron Physician 2017;9:5279–86. 10.19082/5279 29038710 PMC5633226

[15] Koohsari M, Nakaya T, Oka K. Activity-friendly built environments in a super-aged Society, Japan: current challenges and toward a research agenda. Int J Environ Res Public Health 2018;15:2054. 10.3390/ijerph15092054 PMC6163734 30235862

[16] Park S, Zachary WW, Gittelsohn J, et al. Neighborhood influences on physical activity among low-income African American adults with type 2 diabetes mellitus. Diabetes Educ 2020;46:181–90. 10.1177/0145721720906082 32100614 PMC7469716

[17] Cunningham F, Leveno K, Bloom S. Williams obstetrics, 24e. New York, USA: Mcgraw-hill, 2014.

[18] Chasan-Taber L, Chasan-Taber L, Chasan-Taber L, et al. Development and validation of a pregnancy physical activity questionnaire. Med Sci Sports Exerc 2004;36:1750–60. 10.1249/01.MSS.0000142303.49306.0D 15595297

[19] Fathnezhad Kazemi A, Fathnezhad Kazemi A, Fathnezhad Kazemi A, et al. The psychometric properties of the Persian version of the pregnancy physical activity questionnaire. International Journal of Women's Health and Reproduction Sciences 2019;7:54–60. 10.15296/ijwhr.2019.09

[20] Sechrist KR, Walker SN, Pender NJ. Development and psychometric evaluation of the exercise benefits/barriers scale. Res Nurs Health 1987;10:357–65. 10.1002/nur.4770100603 3423307

[21] Amiri Farahani L, Parvizy S, Mohammadi E. The psychometric properties of exercise Benefits. Barriers scale among women. Electronic Physician journal 2017. 10.19082/4780 PMC5586993 28894535

[22] Nascimento SL, Surita FG, Godoy AC, et al. Physical activity patterns and factors related to exercise during pregnancy: a cross sectional study. PLoS One 2015;10:e0128953. 10.1371/journal.pone.0128953 26083416 PMC4470997

[23] Fell DB, Joseph KS, Armson BA, Fell DB, Fell DB, et al. The impact of pregnancy on physical activity level. Matern Child Health J 2009;13:597–603. 10.1007/s10995-008-0404-7 18719984

[24] Berge JM, Larson N, Bauer KW, et al. Are parents of young children practicing healthy nutrition and physical activity behaviors? Pediatrics 2011;127:881–7. 10.1542/peds.2010-3218 21482603 PMC3081185

[25] Antosiak-Cyrak KZ, Demuth A. A study of physical activity levels of pregnant women using the Polish version of pregnancy physical activity questionnaire (PPAQ-Pl). Ginekol Pol 2019;90:250–5. 10.5603/GP.2019.0047 31165463

[26] Santos PC, Abreu S, Moreira C, et al. Impact of compliance with different guidelines on physical activity during pregnancy and perceived barriers to leisure physical activity. J Sports Sci 2014;32:1398–408. 10.1080/02640414.2014.893369 24702128

[27] Hallal PC, Matsudo SM, Matsudo VKR, et al. Physical activity in adults from two Brazilian areas: similarities and differences. Cad Saude Publica 2005;21:573–80. 10.1590/S0102-311X2005000200024 15905920

[28] Leppänen M, Aittasalo M, Raitanen J, et al. Physical activity during pregnancy: predictors of change, perceived support and barriers among women at increased risk of gestational diabetes. Matern Child Health J 2014;18:2158–66. 10.1007/s10995-014-1464-5 24615354

[29] Petrov Fieril K, Fagevik Olsén M, Glantz A, et al. Experiences of exercise during pregnancy among women who perform regular resistance training: a qualitative study. Phys Ther 2014;94:1135–43. 10.2522/ptj.20120432 24786941

[30] Haakstad LAH, Voldner N, Henriksen T, et al. Why do pregnant women stop exercising in the third trimester? Acta Obstet Gynecol Scand 2009;88:1267–75. 10.3109/00016340903284901 19824869

[31] Chasan-Taber L, Schmidt MD, Pekow P, et al. Correlates of physical activity in pregnancy among Latina women. Matern Child Health J 2007;11:353–63. 10.1007/s10995-007-0201-8 17345155

[32] Gaston A, Cramp A. Exercise during pregnancy: a review of patterns and determinants. J Sci Med Sport 2011;14:299–305. 10.1016/j.jsams.2011.02.006 21420359

[33] Mudd LM, Nechuta S, Pivarnik JM, et al. Factors associated with women’s perceptions of physical activity safety during pregnancy. Prev Med 2009;49:194–9. 10.1016/j.ypmed.2009.06.004 19540874

[34] Hinton PS, Olson CM. Predictors of pregnancy-associated change in physical activity in a rural white population. Matern Child Health J 2001;5:7–14. 10.1023/A:1011315616694 11341722

[35] Borodulin K, Sipilä N, Rahkonen O, et al. Socio-Demographic and behavioral variation in barriers to leisure-time physical activity. Scand J Public Health 2016;44:62–9. 10.1177/1403494815604080 26392420

[36] Hegaard HK, Damm P, Hedegaard M, et al. Sports and leisure time physical activity during pregnancy in nulliparous women. Matern Child Health J 2011;15:806–13. 10.1007/s10995-010-0647-y 20680672

[37] et alAmiri Farahani L, Heidari T, Narenji F. Relationship between Pre Menstrual Syndrome with Body Mass Index among University Students. Hayat. 2012; 17 (4) :85-95 URL. Available: http://hayat.tums.ac.ir/article-1-49-en.html

[38] Da Costa D, Ireland K. Perceived benefits and barriers to leisure-time physical activity during pregnancy in previously inactive and active women. Women Health 2013;53:185–202. 10.1080/03630242.2012.758219 23517515

[39] Amiri-Farahani L, Ahmadi K, Hasanpoor-Azghady SB, et al. Development and psychometric testing of the ‘barriers to physical activity during pregnancy scale’ (BPAPS). BMC Public Health 2021;21:1483. 10.1186/s12889-021-11511-3 34325684 PMC8323252

