# Supplementary material for: Exploring the intensity, barriers and correlates of physical activity In Iranian pregnant women: a cross-sectional study
Source: BMJ Open Sport Exerc Med. 2021 Oct 6;7(4):e001020. doi: 10.1136/bmjsem-2020-001020 (PMC8496388; doi:10.1136/bmjsem-2020-001020)
Supplement: Supplementary data [file bmjsem-2020-001020supp001.pdf]

Supplementary material 1: Frequent Distribution of Pregnant Women in Health Centers and Bases in Ilam, Iran, during 2018

**Health centers and bases**	**Frequency**	**Percentage**
Health center number 1	74	24.66
Health center number 4	37	12.33
Health center number 5	25	8.33
Health center number 6	21	7
Health center number 8	21	7
Health center number 9	15	5
Health center number 10	5	1.6
Health center number 13	2	0.06
Health base number 1	48	16
Health base number 3	25	8.33
Health base number 7	27	9
Total	300	100

Supplementary material 2. Frequency distribution of women's demographic and obstetrics history characteristics and comparison of the total score of PA intensity in terms of demographic and obstetrics history characteristics (n = 300)

Characteristics	Category	N (%)	Mean	SD	*P-value
**Age (years)**	≤ 24	85 (28.32)	43.43	26.19	† 0.42
	25-29	98 (32.66)	47.36	27.65	
	30-34	92 (30.66)	49.42	25.76	
	≥ 35	25 (8.33)	50.55	22.33	
**Pre- or early pregnancy BMI (kg/m^2)**	≤ 18.5	12 (4)	35.13	16.23	**† < 0.001**
	18.5-24.9	107 (35.66)	43.77	25.41	
	25-29.9	132 (44)	45.94	23.73	
	≥ 30	49 (16.33)	60.71	31.91	
**Ethnicity**	Fars	11(3.66)	71.42	24.58	**† 0.003**
	Kurdish	256 (85.33)	46.44	25.48	
	Lur	24 (8)	38.80	28.46	
	Lak	9 (3)	59.78	27.85	
**Level of education**	Secondary	21 (7)	33.93	20.46	**† 0.01**
	Diploma	98 (32.66)	44.30	23.54	
	University education	181 (60.33)	50.22	27.67	
**Occupation**	Employed	41 (13.66)	49.03	25.52	‡ 0.62
	Housewife	259 (86.33)	46.85	26.41	
**Income (month's wages: milions Rls)**	Undesirable < 20	34 (11.33)	44.11	29.29	**† 0.02**
	Fairly favourable: 20-40	150 (50)	43.78	23.07	
	Optimal: 40-100	116 (38.66)	52.39	28.48	
**Number of pregnancy**	1	152 (50.66)	43.49	25.52	† 0.09
	2	94 (31.33)	50.29	28.62	
	3	45 (15)	52.62	22.80	
	4	9 (3)	48.78	23.38	
**Number of children**	0	153 (51)	39.73	23.32	**† < 0.001**
	1	112 (37.33)	55.18	28.13	
	2	35 (11.66)	53.84	23.26	
**Gestational age**	10-14	67 (22.33)	37.55	17.44	**† 0.002**

**(wks)**	15-28	123 (41)	48.92	27.87	
	29-37	110 (36.66)	51.01	27.60	
**Participation in childbirth preparation classes**	Yes	34 (11.33)	36.75	16.76	‡ **0.01**
	No	266 (88.66)	48.48	26.97	
**Habit of exercise before pregnancy**	Yes	110 (36.66)	51.11	30.40	‡ **0.04**
	No	190 (63.33)	44.85	23.30	

*Significance level: P < 0.05
†One-way ANOVA test
‡Independent sample t-test

Supplementary material 3. Frequency distribution of women's demographic and obstetrics history characteristics and comparison of the total score of PA barriers in terms of demographic and obstetrics history characteristics (n = 300)

Characteristics	Category	N (%)	Mean	SD	*P-value
**Age (years)**	≤ 24	85 (28.32)	30.83	6.13	† P = 0.86
	25-29	98 (32.66)	30.81	5.39	
	30-34	92 (30.66)	30.33	6.11	
	≥ 35	25 (8.33)	31.36	5.36	
**Pre- or early pregnancy BMI (kg/m^2)**	≤ 18.5	12 (4)	31.75	3.57	† P = 0.15
	18.5-24.9	107 (35.66)	30.08	6.00	
	25-29.9	132 (44)	30.56	5.15	
	≥ 30	49 (16.33)	32.26	7.18	
**Ethnicity**	Fars	11(3.66)	29.72	4.24	† **P = 0.02**
	Kurdish	256 (85.33)	30.70	5.60	
	Lur	24 (8)	33.04	8.19	
	Lak	9 (3)	26.22	2.27	
**Level of education**	Secondary	21 (7)	31.09	8.25	† P = 0.92
	Diploma	98 (32.66)	30.81	6.21	
	University education	181 (60.33)	30.62	5.26	
**Occupation**	Employed	41 (13.66)	31.21	4.20	‡ P = 0.55
	Housewife	259 (86.33)	30.64	6.03	
**Income (month's**	Undesirable < 20	34 (11.33)	29.91	5.33	† **P = 0.01**

**wages: milions Rls)**					
	Fairly favourable: 20-40	150 (50)	31.70	5.69	
	Optimal: 40-100	116 (38.66)	29.68	5.92	
**Number of pregnancy**	1	152 (50.66)	30.85	6.12	**† P = 0.01**
	2	94 (31.33)	29.55	4.86	
	3	45 (15)	31.82	6.31	
	4	9 (3)	35.11	3.95	
**Number of children**	0	153 (51)	30.83	5.93	† P = 0.71
	1	112 (37.33)	30.40	5.66	
	2	35 (11.66)	31.22	5.83	
**Gestational age (wks)**	10-14	67 (22.33)	30.88	5.52	† P = 0.92
	15-28	123 (41)	30.78	6.92	
	29-37	110 (36.66)	30.54	4.53	
**Participation in childbirth preparation classes**	Yes	34 (11.33)	30.61	5.22	‡P = 0.91
	No	266 (88.66)	30.73	5.89	
**Habit of exercise before pregnancy**	Yes	110 (36.66)	29.81	7.03	**‡ P = 0.04**
	No	190 (63.33)	31.24	4.91	

*Significance level: P < 0.05

†One-way ANOVA test

‡Independent sample t-test
